# Insulin resistance indices and coronary risk in adults from Maracaibo city, Venezuela: A cross sectional study

**DOI:** 10.12688/f1000research.13610.2

**Published:** 2018-03-09

**Authors:** Juan Salazar, Valmore Bermúdez, Luis Carlos Olivar, Wheeler Torres, Jim Palmar, Roberto Añez, Maria Gratzia Ordoñez, José Ramón Rivas, María Sofía Martínez, Juan Diego Hernández, Modesto Graterol, Joselyn Rojas

**Affiliations:** 1 Endocrine and Metabolic Research Center, University of Zulia, Maracaibo, Venezuela; 2 Grupo de Investigación Altos Estudios de Frontera (ALEF), Universidad Simón Bolívar, Cucuta, Colombia; 3 Centro de Salud San Marcos, Ministerio de Salud Pública, Provincia de Santa Elena, Ecuador; 4 Pulmonary and Critical Care Medicine Department, Brigham and Women’s Hospital, Harvard Medical School, Boston, MA, USA

**Keywords:** insulin resistance, coronary risk, triglycerides, HOMA2-IR, HDL-C

## Abstract

**Background:** Insulin resistance (IR) is a metabolic disorder related to atherosclerosis. Its measurement is of great importance not only as a marker of diabetes but also for cardiovascular disease. The aim of this research study was to evaluate the relationship between various IR indices and coronary risk in an adult population from Maracaibo city, Venezuela.

**Methods:** The Maracaibo City Metabolic Syndrome Prevalence Study is a descriptive, cross-sectional study with random and multi-stage sampling. In this sub study, 1272 individuals of both genders were selected with the measurement of basal insulin and coronary risk according to the Framingham-Wilson formula calibrated for our population. The insulin resistance indices evaluated were HOMA2-IR, triglycerides and glucose index (TyG) and triglycerides/HDL ratio (TG/HDL). The predictive capacity and association between each index and the coronary risk event in 10 years were determined.

**Results: **Of the evaluated population, 55.2% were female, 34.8% had a coronary risk ≥5% in 10 years, with the TG/HDL and TyG indices showing the highest AUC 0.712 (0.681-0.743) and 0.707 (0.675-0.739), respectively; compared to HOMA2-IR. Both were also the indices most associated with increased coronary risk, especially TG/HDL ≥3 with a higher association [OR = 2.83 (1.74-4.61); p<0.01] after multivariable adjustment.

**Conclusions:** TyG (≥4.5) and TG/HDL (≥3) indices showed a great predictive capacity of higher coronary risk, with being TG/HDL more associated even after adjusting for abdominal obesity and hs-CRP. Therefore, these represent useful tools for determining IR.

## Introduction

Insulin resistance (IR) is defined as a metabolic disorder in which the insulin effects in the target tissue are diminished, leading to derangements in carbohydrate, lipid and protein metabolism ^1^. IR has been related to the development of several pathologies such as type 2 Diabetes Mellitus (DM2) ^2^, Metabolic Syndrome (MS) ^3^ and neurodegenerative diseases ^4^. Therefore, the diagnosis and treatment of IR before the expression of the clinical disease are highly important for clinicians ^5^. However, most methods to evaluate IR are costly and difficult to operate, such as the euglycemic-hyperinsulinemic clamp, which is considered the gold standard method for its detection ^6^.

There is a need to implement new markers that are easier to interpret and more affordable, one being the Homeostasis Model Assessment (HOMA-IR), one of the most widely used, which is calculated from the measurement of fasting glucose and insulin concentration in the plasma ^7^. However, the need to determine plasma insulin is a characteristic that limits its use in low-income populations. This limitation has been curbed by the use of the Triglycerides-Glucose (TyG) index, which is obtained from the fasting product of plasma glucose and triglycerides (TG) levels, and has shown an excellent predictive capacity to determine IR even in 2004 subjects of both genders ≥18 years from Maracaibo city in a previous report of our research team ^8^.

The potential role of IR in the development of cardiovascular diseases (CVD) as a consequence of metabolic derangements, such as hyperglycemia, dyslipidemia, and inflammation together with hypertension, are involved in the progression of atherosclerosis ^9^. The aim of this study was to evaluate the relationship between IR indices and coronary risk in an adult population from Maracaibo city, Venezuela.

## Methods

### Ethical considerations

The study was approved by the Bioethics Committee of the Endocrine and Metabolic Research Center – University of Zulia (approval number: BEC-006-0305). This ethical approval included all future studies that used the data from the Maracaibo City Metabolic Syndrome Prevalence Study (MMSPS). All participants signed written consent for participation in the study before being questioned and physically examined by a trained team.

### Study design and population selection

The MMSPS is a descriptive, cross-sectional study performed in Maracaibo city, Venezuela, during the period May 2007 - December 2009, with the aim to determine the prevalence of MS and cardiovascular risk factors in the adult population of this county. The study include a total of 2230 individuals of both gender, older than 18 years old, and the study protocol was previously reported ^10^. The most important aspects of the protocol are presented here. Maracaibo city was divided into parishes, which were sampled proportionally through a multistage random sampling, defining conglomerates in two phases: In the first phase, the conglomerates represented the sectors of the 18 parishes, selecting 4 areas per parish by means of simple random sampling; in the second phase, the conglomerates were represented by the neighborhood of each chosen area, to which a random number was assigned.

For this present sub-analysis, individuals between 30–74 years of age were included, excluding those with a past medical history of ischemic heart disease (requirements necessary for the calculation and calibration of the Framingham-Wilson equation), as well as those individuals without available basal insulin determination necessary for HOMA2-IR calculation, thus the final total sample was 1272 individuals.

### Evaluation of individuals

All individuals were evaluated, with a complete clinical history performed by trained assistants. Personal past medical and family history were gathered, with an emphasis on metabolic, endocrine and cardiovascular diseases history, as well as sociodemographic characteristics, such as age, gender and race.

### Laboratory analysis

After 8 hours of fasting, determination of glucose, total cholesterol, TG, and HDL-C was done with an automated analyzer (Human Gesellschaft fur Biochemica und Diagnostica mbH, Germany). The intra-assay coefficient of variation for total cholesterol, TG, glucose and insulin was 3%, 5%, 3%, and <10%, respectively. Insulin was determined using an ultrasensitive ELISA double-sandwich method (DRG Instruments GmbH, Germany), with a detection limit <1 mU/L. Likewise, serum hs-CRP levels were quantified employing immunoturbidimetric assays (Human Gesellschaft für Biochemica and Diagnostica, mH).

### IR indices

The calculation of the Triglycerides and Glucose index (TyG) was performed according to Simental-Mendía *et al*. ^11^, using the equation = ln [fasting triglycerides (mg / dl) × basal glucose (mg / dl)/2]; thus expressing itself on a logarithmic scale. Previously, we proposed for a 4.5 cut-off point for this index to define IR for this population ^8^. The Triglycerides/HDL ratio (TG/HDL) was calculated from the division of TG between HDL-C; 3 was used as a as cut-off point ^12^. The HOMA2-IR was calculated using the HOMA-Calculator v2.2.2 software provided by the Oxford Center for Diabetes Endocrinology and Metabolism. The proposed cut-off point for our population was 2.00 to define IR ^13^.

### Definitions

For the evaluation of coronary risk, the Framingham-Wilson formula calibrated for the population of Maracaibo was used ^14^. The coronary risk was classified into two categories: 1) <5% in 10 years; 2) ≥5% in 10 years. Type 2 diabetic patients were defined with one of the following criteria: a) previous diagnosis of DM2; b) no previous personal history of DM2, but with fasting glucose greater than or equal to 126 mg/dl in two different measurements ^15^. Abdominal obesity was defined according to the cut-off points for abdominal circumference previously obtained in our population (Women: 91cm; Men: 98cm) ^16^, as well as the levels of high Sensitivity C-Reactive Protein (hs-CRP), which were measured randomly in 842 subjects, elevated hs-CRP defined as ≥0.765 mg/L ^17^.

### Statistical analysis

Qualitative variables were expressed in absolute and relative frequencies, evaluating the association between them using the χ ^2^ test (Chi square). Quantitative variables were expressed as arithmetic means±SD, after analysis of normality by Geary test. The variables with non-normal distribution were subjected to logarithmic transformation.

ROC curves were constructed to analyze the predictive capacity of coronary risk of IR index. ROC curves were constructed for the total and specific sample by gender using R software version 3.4.1. The Area Under the Curve (AUC) was used to establish the predictive ability of the IR indices, where an AUC of 1.00 is considered a perfect diagnostic test ^18^. The comparisons between AUC were made through the Delong’s test ^19^. Likewise, a logistic regression model was performed for which index was associated with a coronary risk ≥5% (dependent variable), adjusted by age group, sex and IR indices. In successive models, these were adjusted according to abdominal obesity and hs-CRP. All data were analyzed with SPSS v.21 for Windows (IBM Chicago, IL). The results were considered statistically significant when p<0.05.

## Results

### General characteristics of the sample

A total of 1272 individuals were evaluated, 55.2% (n=702) were female; 65.2% (n=829) had coronary risk <5% in 10 years, while 34.8% (n=443) had coronary risk ≥5% in 10 years. The frequency of DM2 for the two coronary risk groups was 29.6% (n=45) and 70.4% (n=107), respectively. The mean age of the sample population was 47.13±10.92 years. Other general characteristics of the sample population are presented in Table 1.

**Table 1.** General characteristics of the sample population from Maracaibo city, Venezuela, according to coronary risk.

	<5% Coronary risk (n=829)	≥5% Coronary risk (n=443)
**Gender (n; %)**		
Female	499; 71.1	203; 28.9
Male	330; 57.9	240; 42.1
**Type 2 diabetes mellitus** **(n; %)**		
Absent	784; 70.0	336; 30.0
Present	45; 29.6	107; 70.4
**Abdominal obesity (n; %)**		
Absent	378; 75.1	125; 24.9
Present	451; 58.6	318; 41.4
**hs-C Reactive Protein** (mg/L) * (n; %)		
Normal	403; 66.9	199; 33.1
Elevated	135; 56.3	105; 43.7
**Age (Mean±SD)**	42.29±8.54	56.19±8.94
**TyG (Mean±SD)**	4.61±0.31	4.89±0.35
**HOMA2-IR (Mean±SD)**	2.17±1.43	2.54±1.55
**TG**/ **HDL (Mean±SD)**	3.11±2.54	5.55±5.81

*High sensititve C-reactive protein values were measured in n=842. TyG: Tryglycerides-glucose index; TG/HDL: Tryglycerides/HDL ratio; SD: Standard deviation.

### Insulin resistance index and coronary risk

**Figure 1.**
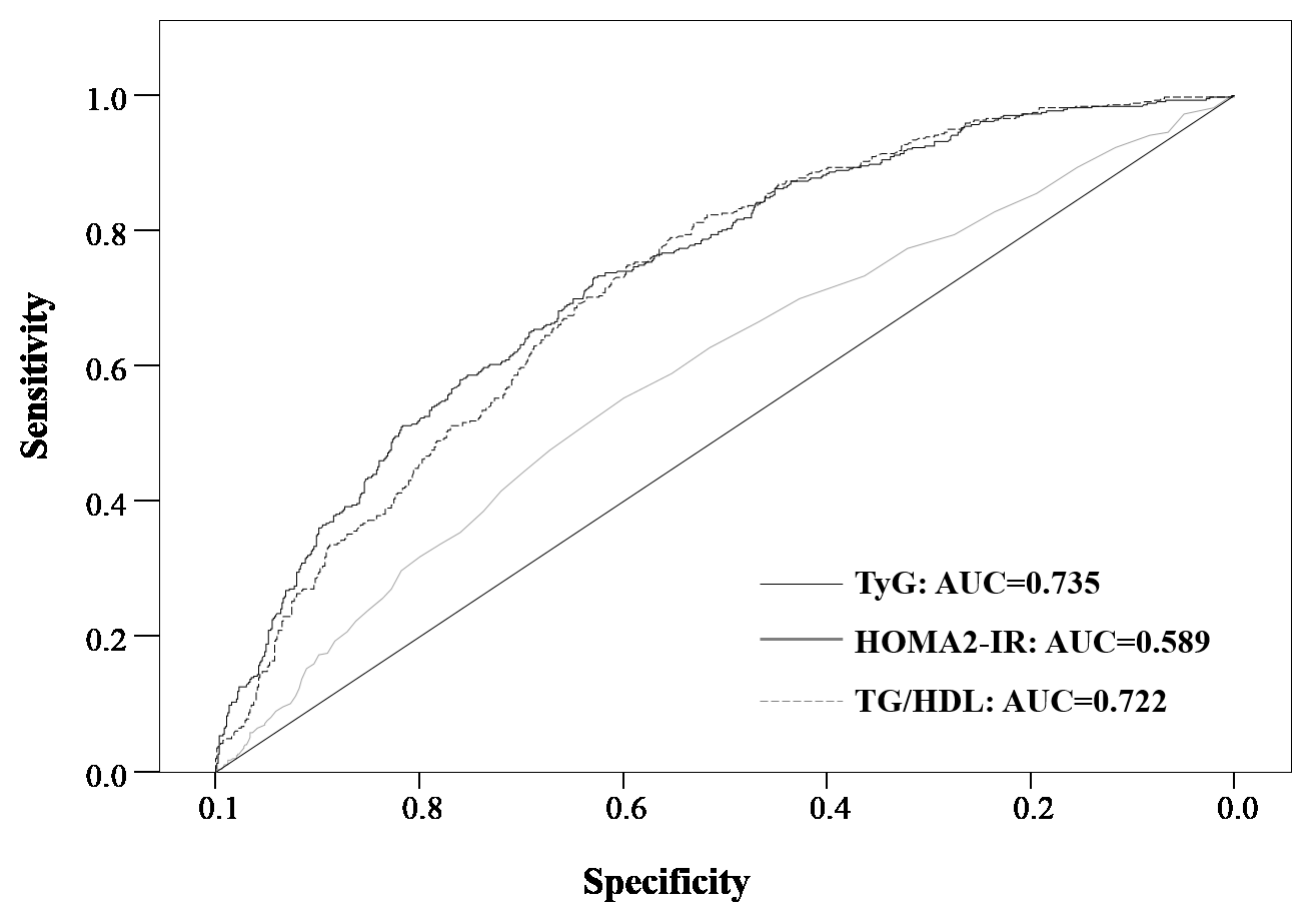
ROC curves for insulin resistant indices in a population from Maracaibo city, Venezuela. AUC, area under the curve; TyG: Tryglycerides-glucose index; TG/HDL: Tryglycerides/HDL ratio.

When evaluating the predictive capacity of the IR indices for coronary risk ( Figure 1), it was evidenced that the TyG index had an AUC=0.735 (CI95%: 0.707-0.763), higher than that observed for the HOMA2-IR [(AUC=0.589; CI95%: 0.556-0.622; p=2.2×10 ^-26^)]. Likewise, the TG/HDL index showed a greater predictive capacity compared to HOMA2IR (AUC=0.772 vs. AUC=0.589; p=1.20×10 ^-12^). There were no significant differences in the predictive capacity between the TyG index and the TG/HDL (p=0.079), ( Table 2).

**Table 2.** Comparison of AUC obtained from each insulin resistance index for the sample population from Maracaibo city, Venezuela.

	TyG (A)	HOMA2-IR (B)	TG/HDL (C)	DeLong Test		
	AUC (CI95%)	AUC (CI95%)	AUC (CI95%)	A vs B	A vs C	B vs C
**All**	0.735 (0.707-0.763)	0.589 (0.556-0.622)	0.722 (0.694-0.750)	2.2×10 ^-16^	0.079	1.20×10 ^-12^
Female	0.770 (0.734-0.807)	0.590 (0.543-0.637)	0.767 (0.730-0.803)	1.68×10 ^-12^	0.697	9.19×10 ^-11^
Male	0.682 (0.638-0.726)	0.578 (0.531-0.626)	0.649 (0.604-0.694)	8.03×10 ^-5^	0.005	0.009
*Individuals without Diabetes mellitus*						
**All**	0.707 (0.675-0.739)	0.563 (0.527-0.600)	0.712 (0.681-0.743)	4.12×10 ^-12^	0.447	1.24×10 ^-12^
Female ^a^	0.746 (0.704-0.787)	0.570 (0.518-0.622)	0.759 (0.719-0.798)	5.51×10 ^-9^	0.174	5.81×10 ^-10^
Male ^a^	0.648 (0.598-0.698)	0.549 (0.497-0.602)	0.637 (0.587-0.687)	8.76×10 ^-4^	0.276	0.003

AUC, area under the curve; TyG: Tryglycerides-glucose index; TG/HDL: Tryglycerides/HDL ratio; CI: Confidence Interval ^a^Delong’s test between female and male gender TyG = 0.002; HOMA2-IR = 0.729; TG/HDL = 7.86×10 ^-5^

In the evaluation by gender, both women and men had a TyG index with significantly higher AUC than HOMA2-IR (Women: AUC=0.770 vs AUC=0.590; p=1.68×10 ^-12^; Men: AUC=0.682 vs AUC=0.578; p=8.03×10 ^-5^), whereas only in men the TyG index had a statistically greater predictive capacity than the TG/HDL index (AUC=0.682 vs. AUC=0.649; p=0.005). Likewise, in women a greater predictive capacity compared to men was observed for both the TyG index (AUC=0.770 vs AUC=0.682; p=0.002), and TG/HDL (AUC=0.767 vs. 0.649; p=7.86×10 ^-5^) ( Table 2).

### IR indices and coronary risk in individuals without DM

**Figure 2.**
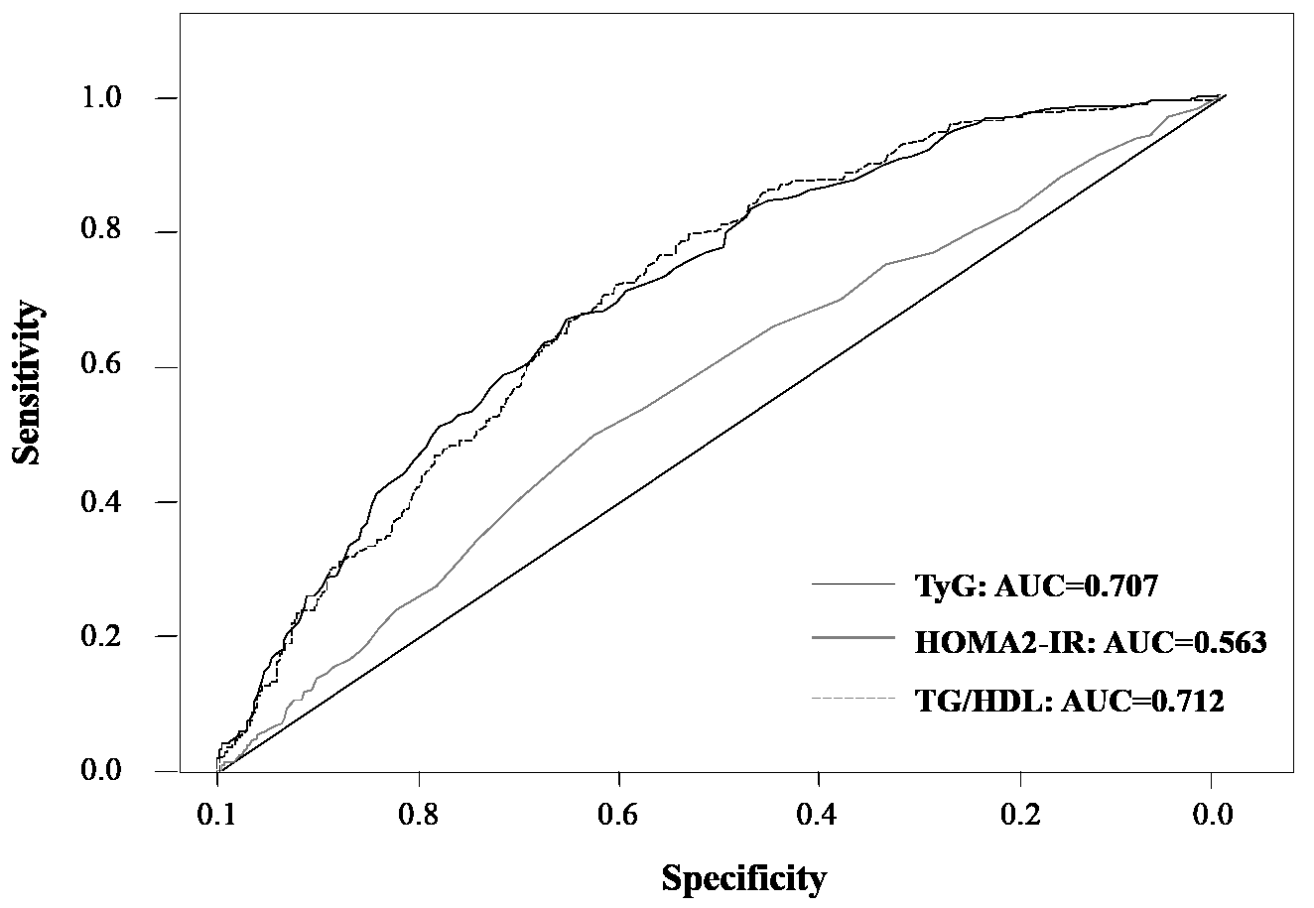
ROC curve for insulin resistant index in individuals without diabetes mellitus in a population from Maracaibo city, Venezuela. AUC, area under the curve; TyG: Tryglycerides-glucose index; TG/HDL: Tryglycerides/HDL ratio.

Similarly, when evaluating the indices in individuals without diagnosis of DM2 ( Figure 2), it was demonstrated that TG/HDL and TyG indices were the greatest predictors of individuals without DM2, with AUC values of 0.712 (CI95%: 0.681-0.743) and 0.707 (CI95%: 0.675-0.739), respectively. These were significantly superior to those exhibited by HOMA2-IR, even in the analysis by gender ( Table 2).

### IR indices and coronary risk

During the evaluation of subjects’ distribution according to IR indices and coronary risk in 10 years ( Table 3), a statistically significant association was found. However, it was observed in the multivariate model that only individuals with a TG/HDL ≥3 were more likely to present ≥5% coronary risk in 10 years (OR: 3.17 CI95%: 2.15-4.68; p<0.01), even after model adjusting for abdominal obesity and hs-CRP (OR: 2.83 CI95%: 1.74-4.61; p<0.01).

**Table 3.** Distribution of individuals according to insulin resistance indices and coronary risk in a sample population from Maracaibo city, Venezuela.

	<5% coronary risk		≥5% coronary risk		χ ^2^ ( *p) ^a^*	Model 1 OR (IC95%); *p*	Model 2 OR (IC95%); *p*	Model 3 OR (IC95%); *p*
	n	%	n	%				
**TyG**					98.55 (<0.001)			
<4.5	302	36.4	46	10.4		1	1	1
≥4.5	527	63.6	397	89.6		1.86 (1.15-3.02); 0.01	1.82 (1.12-2.96); 0.02	1.48 (0.82-2.67); 0.19
**HOMA2-IR**					23.17 (<0.001)			
<2	458	55.2	182	41.1		1	1	1
≥2	371	44.8	261	58.9		1.21 (0.89-1.65); 0.22	1.09 (0.79-1.51); 0.56	1.17 (0.79-1.73); 0.43
**TG/HDL**					128.43 (<0.001)			
<3	527	63.6	134	30.2		1	1	1
≥3	302	36.4	309	69.8		3.17 (2.15-4.68); <0.01	3.01 (2.04-4.46); <0.01	2.83 (1.74-4.61); <0.01
**Total**	**829**	**100.0**	**443**	**100.0**				

TG: triglycerides; TyG: triglycerides-glucose index. ^a^Pearson’s chi-square test Model 1: adjusted by indices of insulin resistance, age groups and sex. Excluding diabetic subjects Model 2: Model 1 + abdominal obesity Model 3: Model 2 + Elevated hs-CRP

MMSPS Insulin resistance indices and coronary risk dataset BMI: Body Mass Index, WaistC: Waist Circumference, BP: Blood Pressure; hs-CRP: high Sensitivity C Reactive Protein. Click here for additional data file. Copyright: © 2018 Salazar J et al. 2018 Data associated with the article are available under the terms of the Creative Commons Zero "No rights reserved" data waiver (CC0 1.0 Public domain dedication).

## Discussion

IR is currently well-known for its physiopathological role in conditions such as obesity, arterial hypertension, dyslipidemia, DM2 and CVD ^2, 3, 9^. The detection of IR in primary care is a potential prevention strategy. For this reason, the use of low cost and easy use indices, such as TyG and TG/HDL, could be valuable tools in daily clinical practice, even over HOMA2-IR.

Different physiopathological mechanisms has been linked to IR, including an increase in cardiovascular risk by promoting endothelial dysfunction, inhibiting the synthesis of nitric oxide, and stimulating the secretion of endothelin-1 and the expression of cell adhesion molecules ^20^. It also affects the smooth muscle cells by promoting proliferation, migration and apoptosis, making the atherosclerotic plaque more unstable, which is associated with secretion of pro-inflammatory mediators by macrophages ^21^. These morphological and functional changes in the vascular wall and the heart could explain the increased cardiovascular risk in IR states ^9^.

Several studies have evaluated the predictive capacity of TyG index for CVD, but there are few reports for Latin American populations. In this analysis, it was found that TyG is a better predictor of coronary risk compared with HOMA2-IR. Similarly, Martínez-Larrad *et al*. ^22^, in a combined analysis conducted in non-diabetic individuals from the Mexico City Diabetes Study, the San Antonio Heart Study and Spanish Insulin Resistance Study, observed that the TyG index had higher AUC (between 0.673-0.875) to discriminate ≥10% risk coronary in 10 years than the HOMA-IR (between 0.579-0,746), as well as other less used IR indices, such as the McAuleys index and the insulin sensitivity index of Avignon.

This behavior has also been determined in two Asian populations, where the TyG index was a better predictor of coronary arterial calcification than the HOMA-IR ^23, 24^, although in one of these populations, it was observed that by including another predictive variable, such as the abdominal circumference, the predictive capacity of this marker increased ^24^. On the other hand, Sánchez-Íñigo *et al*. ^25^ showed that in 5014 patients belonging to The Vascular Metabolic CUN cohort, the association of the TyG index categorized in quintiles and the risk of developing a coronary event was twice the risk in the upper quintile. In addition, Irace *et al*. ^26^ analyzed the relationship between the TyG and HOMA indices with the risk of carotid atherosclerosis, and demonstrated that only the former was significantly associated, even after adjustment with the presence of metabolic syndrome.

Our results coincide with these previously reported findings that reveal a significant superiority of TyG over HOMA2-IR in the prediction of coronary risk, which was also more marked in the female population, probably associated with a greater distribution of intra-abdominal fat and proatherogenic factors. This interaction between the proinflammatory molecule and the TyG was evident in multivariable analysis after the adjustment by hs-CRP.

In regards to the TG/HDL ratio, most studies in Latin America have compared the predictive capacity for cardiovascular risk or association with cardiovascular risk factors in children ^27^. Only those reports of Salazar *et al*. ^28, 29^ in the Argentinean population have linked the TG/HDL ratio with cardiovascular events in Latin American adults. In this sense, our findings coincide with those shown in the Argentinean population, evidencing not only the high predictive capacity of individuals with coronary risk ≥5% in 10 years by the TG/HDL ratio, but the close relationship that exists between the variables even after adjustment for covariates such as abdominal obesity and hs-CRP, higher than the TyG index. These results differ from those exhibited by Du *et al*. ^30^ in more than 7000 Chinese individuals, who showed that TyG index is the best predictor of IR, whereas TG/HDL ratio is body fat distribution dependent.

The TyG and TG/HDL indices superiority over HOMA2-IR in the prediction of coronary risk demonstrates the importance of serum TG in the pathophysiology of IR. Although one of the disadvantages posed for HOMA-IR is that it only reflects IR in the liver, given the ability of basal insulin to suppress gluconeogenesis in this organ during the fasting period ^26^. In this study, the mathematical model HOMA2-IR was used, whose advantage with its predecessor is the estimation of peripheral resistance to insulin ^31^. Therefore, the greater prediction of the indices that have serum TG in their calculation does not depend specifically on the ability to measure peripheral IR in skeletal muscle or adipose tissue, but possibly, the greater contribution of TG in the development of IR ^32^.

Additionally, the differences in regards of IR according to gender and its potential influence on cardiovascular risk is a controversial topic in the endocrinology field ^33^. Our findings reflect that this difference is shown even in the measurement methods, with a greater predictive capacity of coronary risk in women, possibly associated with differences in serum levels of TG and HDL-C according to gender, an aspect that must be taken into consideration when making determinations.

It should be noted that because of the cross-sectional design of the study, causality cannot be defined, so future prospective design studies should evaluate these IR indices for the prediction of cardiovascular mortality. However, our findings demonstrate the importance of the TyG and TG/HDL indices, especially the latter in the prediction of coronary risk, and these are tools to determine IR of easier application in regions where the economic aspect is a limitation of measurement for serum insulin.

## Data availability

The data referenced by this article are under copyright with the following copyright statement: Copyright: © 2018 Salazar J et al.

Data associated with the article are available under the terms of the Creative Commons Zero "No rights reserved" data waiver (CC0 1.0 Public domain dedication). 

**Dataset 1: MMSPS Insulin resistance indices and coronary risk dataset.** BMI: Body Mass Index, WaistC: Waist Circumference, BP: Blood Pressure; hs-CRP: high Sensitivity C Reactive Protein. DOI, 10.5256/f1000research.13610.d189587 ^34^
