# Supplementary material for: Insulin resistance indices and coronary risk in adults from Maracaibo city, Venezuela: A cross sectional study
Source: F1000Res. 2018 Mar 9;7:44. Originally published 2018 Jan 11. [Version 2] doi: 10.12688/f1000research.13610.2 (PMC6107995; doi:10.12688/f1000research.13610.2)
Supplement: Peer review history

# Peer review: Referee response for version 2

**DOI:** 10.5256/f1000research.15459.r36517

**Published:** 2018-08-23

**Authors:** Anirban Sinha

**Affiliations:** 1 Department of Endocrinology and Metabolism, Medical College and Hospital, Kolkata, India

This article is acceptable to me for indexing. Background of this study is very important as indices of insulin resistance is changing very fast and all of them may not be sensitive for different population throughout the world.

 Study method is convincing and results are acceptable. 34.8% had coronary risk of >5% in 10 yrs which is reflective of population in real time. Tg/HDL and TyG indices were statistically Higher AUC than HOMA2-IR. 

 Overall this is a acceptable study which can be reproduced in different populations of the world.

I have read this submission. I believe that I have an appropriate level of expertise to confirm that it is of an acceptable scientific standard.

# Peer review: Referee response for version 2

**DOI:** 10.5256/f1000research.15459.r31726

**Published:** 2018-03-22

**Authors:** Miguel Murguía-Romero

**Affiliations:** 1 Iztacala School of Graduate Studies, National Autonomous University of Mexico (UNAM), Tlalnepantla, Mexico

The article has been improved by the corrections made by the authors.

 I have only one suggestion. According to the calculations with the dataset used in the analysis (“Dataset 1: MMSPS Insulin resistance indices and coronary risk dataset”), the formula used by the authors is the one indicated by Salazar et al. 2017, as it is actually indicated in the text that cites reference 8: "ln [Fasting triglyceride (mg / dl) x Fasting glucose (mg / dl)] / 2"

 So, that is the formula that should be included in the text of the article (version 2).

 Incorrectly, the formula is written in the text as: "ln [Fasting triglyceride (mg / dl) x Fasting glucose (mg / dl) / 2]", as reported by Simental-Mendía et al. 2008.

I have read this submission. I believe that I have an appropriate level of expertise to confirm that it is of an acceptable scientific standard.

# Peer review: Referee response for version 1

**DOI:** 10.5256/f1000research.14784.r30640

**Published:** 2018-02-22

**Authors:** Miguel Murguía-Romero

**Affiliations:** 1 Iztacala School of Graduate Studies, National Autonomous University of Mexico (UNAM), Tlalnepantla, Mexico

The article “Insulin resistance indices and coronary risk in adults from Maracaibo city, Venezuela: A cross sectional study”, supports the use of TyG and TG/HDL formulas as a reliable way to estimate the coronary risk in a Venezuelan population, contributing with the use of these indices in the study of Latin American populations.

 This article is valuable specifically because: 

- Addresses the research problem in an appropriate way using the Framingham-Wilson formula to evaluate coronary risk, and including relevant IR indices in the analysis.
- The results are valuable because the analysis by gender is included, as well as ROC curves are used to estimate the predictive capacity of the indices.
- The statistical analysis is well applied: the descriptive statistical is informative and contribute to adequately contextualize the sample characteristics; also the chosen tests, and the statistical parameter reported are adequate.
- The supplementary material is well structured and the data are congruent with the text (“General characteristics of the sample”) and with Table 1 (according to a rapid analysis I made importing the file into a database).

There are, however, several inconsistencies that may be corrected to improve it:

1. An inconsistency is evident in the formula of TyG; Simental et al. (2008) cite the TyG formula as:
  ln[fasting triglycerides (mg/dL) x fasting glucose (mg/dL)/2]
  While this article cite TyG as:
  ln[fasting triglycerides (mg/dL) x fasting glucose (mg/dL) ]/2
  Are the differences between formulas only a typing error? I think authors have an opportunity to clarify which one of this formulas makes better sense for biomedical research along with the relationships between the physiological parameters included in this index.
2. At the header of Table 1, an “a” is missing in “Maracaibo”.
3. There is no correspondence between the declared quantity of participants with coronary risk bigger than 5% in the text  (n=448; Page 4, 2nd column, 1st line) vs. the one reported in Table 1 (n=443)
4. The range of cell 1 in Table 1, written as “0.735 (0.707-0.63)”, is incongruent with the one reported in the text. Authors must clarified which one is the correct one.
5. Table 3, first column: “TG/HDL” instead “TAG/HDL”. Please, review it for homogeneity also in “Discussion”.

I have read this submission. I believe that I have an appropriate level of expertise to confirm that it is of an acceptable scientific standard, however I have significant reservations, as outlined above.
